# Practices and Components Associated With Woman-Centered Obstetric Care in a Population in Mexico: A Cross-Sectional Study

**DOI:** 10.7759/cureus.104286

**Published:** 2026-02-26

**Authors:** Zenon Morales-Orozco, Javier García-Olan, Sergio Quiroz-Gomez, Karla del Socorro Celorio-Méndez

**Affiliations:** 1 Department of Health Services, Servicios de Salud del Instituto Mexicano del Seguro Social para el Bienestar (IMSS-BIENESTAR), Villahermosa, MEX; 2 Division of Health Sciences, Juárez Autonomous University of Tabasco, Villahermosa, MEX

**Keywords:** informed consent, maternal health services, obstetric violence, patient-centered care, woman-centered care

## Abstract

Introduction: Obstetric violence (OV) constitutes a form of violation of women's human rights and affects the quality of maternal health care. The objective of this study is to identify manifestations of obstetric violence in the Health Services Network of the Nacajuca Health Jurisdiction, Tabasco, Mexico, and to determine the factors associated with adequate obstetric care.

Methodology: A descriptive-observational, cross-sectional study was conducted on 268 participants, including 216 postpartum women and 52 health professionals. The Woman-Centered Obstetric Care Index (WCOCI) was applied in accordance with the provisions set forth in NOM-007-SSA2-2016. Frequencies, chi-squared tests, and Pearson's correlation were calculated. Prevalence ratios (PRs) were estimated using Poisson models with robust variance, with 95% confidence intervals (CIs).

Results: Of the patients, 87.5% received adequate care, while 12.5% received inadequate care. The factors associated with adequate care were as follows: close monitoring (PR=1.72, 95% CI: 1.30-2.28), explanation of procedures (PR=1.55, 95% CI: 1.20-2.00), prior authorization for vaginal examination (PR=1.40, 95% CI: 1.05-1.87), accompaniment by a family member (PR=1.32, 95% CI: 1.01-1.70), and respect for the time needed (PR=1.28, 95% CI: 1.00-1.65).

Conclusions: While the majority of women received adequate obstetric care, there is a persistent prevalence of practices that contravene established standards of respectful care, particularly concerning informed consent and privacy. The implementation of continuous training strategies and woman-centered care protocols is imperative.

## Introduction

Globally, over the last two decades, the focus on maternal health has shifted from a paradigm centered on service coverage to one that prioritizes quality and care, seeking to make childbirth a positive, dignified experience, free from fear and pain [1].

Despite the efforts made, reality remains far from ideal. According to Bohren et al., 55% of women receiving care during childbirth experience at least one act of obstetric violence (OV), with the most prevalent manifestation being lack of informed consent [2]. OV is defined as "a specific form of violence perpetrated by health professionals (predominantly doctors and nurses) against pregnant women, women in labor, and women who have recently given birth that constitutes a violation of women's reproductive and sexual rights" [3]. The consequences of this malpractice include increased pain, an increase in the number of future elective cesarean sections, post-traumatic stress disorder, and a decrease in exclusive breastfeeding. Conversely, OV has been demonstrated to diminish the level of trust in health services and result in a delay in seeking care, consequently increasing maternal morbidity and mortality [4].

The state of Tabasco presents a complicated scenario, as recent data indicate that it remains among the highest in the country in terms of maternal mortality, with almost 14 deaths per 10,000 live births in 2020, which is a major problem [5].

In an effort to raise awareness of this problem and establish regulations to address it, the General Law on Women's Access to a Life Free of Violence [6] and NOM-007-SSA2-2016 [7] have been implemented. These legal documents establish minimum criteria for accompaniment, informed consent, non-pharmacological analgesia, and the elimination of routine practices without clinical indication. However, an examination of official reports on the implementation of these provisions reveals considerable gaps in infrastructure, training, and protocol adoption, especially in rural and semi-urban contexts. In relation to this, the Encuesta Nacional de la Dinámica de los Hogares (ENDIREH) 2021 [8] reported that 36% of women who gave birth or had a cesarean section between 2016 and 2021 experienced some form of abuse, with the indigenous population being the most affected group.

Objective

We aim to characterize woman-centered obstetric care after pregnancy (postpartum period) and analyze its determinants in the municipality of Nacajuca, to identify critical aspects that require improvement actions, and generate evidence that exposes the current health situation in relation to this issue.

## Materials and methods

A descriptive-observational, cross-sectional study was conducted to characterize the quality and patient-centeredness of intrapartum and immediate postpartum care within the Health Services Network of the municipality of Nacajuca, Tabasco, comprising 15 primary care health centers and one general hospital, from January to December 2023.

The study population comprised healthcare personnel (e.g., doctors and nurses) from the healthcare network who had been working for at least six months, as well as patients who were already in the immediate postpartum period (physiological or surgical) and were clinically stable.

The sample size was calculated for an expected proportion of obstetric violence of 30%, a confidence level of 95%, and an absolute precision of 5%, resulting in 216 patients. The final sample also included 52 health professionals, who were selected for convenience.

An instrument was employed to ascertain the sociodemographic and occupational characteristics of healthcare personnel.

The Woman-Centered Obstetric Care Index (WCOCI) instrument was developed to assess patients' perceptions of the care they received and thus operationalize factors that could be related to the presence of obstetric violence. To ensure patient comfort and an appropriate mental and physical state when answering questions, the instrument was administered once patients had had at least six hours after delivery, striving to be as specific as possible and taking into account patients' doubts to avoid obtaining misleading and inaccurate data. This instrument was created based on NOM-007-SSA2-2016, and it contains 12 items and a scoring system from 0 to 12 (0/1 per item). The care provided to patients is classified as poor, fair, good, or excellent, depending on the result obtained. Conversely, in the context of the association analysis, cases categorized as good/excellent were dichotomized as adequate care, while those classified as poor/fair were dichotomized as inadequate care.

The reliability of the WCOCI instrument was assessed using Cronbach's α (0.82) and sample adequacy using the Kaiser-Meyer-Olkin (KMO) test (0.78) and Bartlett's test (p<0.001).

We evaluated the distribution of each WCOCI item and examined which care process components were most strongly associated with the overall adequacy classification. Covariates considered for adjustment included age, marital status, indigenous status, and mode of delivery, selected a priori based on plausibility as potential confounders.

Pearson's chi-squared test was employed to analyze the association between factors and adequate care (WCOCI). Prevalence ratios (PRs) with 95% confidence intervals (CIs) were estimated using Poisson models with robust variance, adjusting for age, marital status, indigenous status, and type of delivery. The data were captured and analyzed using the SPSS statistical software package version 27 (IBM Corp., Armonk, NY).

Ethical considerations

The research was conducted in accordance with the principles of the Declaration of Helsinki and applicable national regulations.

The comprehensive protocol was subjected to a thorough evaluation and received unanimous approval from the Research Committee of the Academic Division of Health Sciences, Juárez Autonomous University of Tabasco (approval number: 181, 2025).

All participants provided written informed consent; clinical stability was verified, and it was ensured that participation would not interfere with care. The data were collected anonymously and subsequently coded before being stored on equipment with restricted access.

## Results

A total of 268 subjects were surveyed, including healthcare personnel and/or midwives (52 participants) and postpartum women (216 participants).

Of the healthcare personnel, 78.8% (n=41) were women, while the remaining percentage (21.2%) were men. The most prevalent age range was 20-29 years, comprising 71.2% of the total sample (n=37). The demographic composition of the sample is as follows: 23.1% of the participants were between the ages of 30 and 39, and 5.7% were between the ages of 40 and 49.

Of the staff members, 84.6% (n=44) possessed a bachelor's degree, 9.6% (n=5) had completed postgraduate studies, and 5.8% (n=3) indicated that their degree was in a technical field related to health.

A subsequent analysis of the hospital positions held by the staff revealed that 57.7% (n=30) of the positions were held by doctors, 25% (n=13) by nurses, 9.5% (n=5) by paramedics or social service interns, and finally, 7.7% (n=4) by midwives.

The maximum reported duration of professional experience was 20 years (n=1). However, the majority of the staff (82.7%) possessed between 0 and 4 years of experience.

In regard to the subject's workload, the range of weekly hours worked was from eight to 82 hours. It was observed that 40 hours was the most prevalent number of hours worked, constituting 69.2% (n=36) of the sample of workers. While 78.8% (n=41) of staff members did not self-report experiencing excessive workload, 55.8% reported at least one physical, intellectual, or emotional disorder secondary to work.

A mere 5.8% (n=3) of the participants indicated that they had undergone training or education in the area of obstetric violence within the previous two years.

In consideration of female users, an analysis of sociodemographic and obstetric data (Table 1) reveals that the predominant proportion of users falls within the 20-29 age range, with significant follow-up by the 30-39 and 10-19 age brackets.

**Table 1:** Sociodemographic and obstetric data

Sociodemographic and obstetric data	Frequency	%
Patient's age		
10-19 years	36	16.7
20-29 years	113	52.3
30-39 years	62	28.7
40-49 years	5	2.3
Marital status		
Married	50	23.1
Single	13	6
Common-law marriage	152	70.4
Widowed	1	0.5
Type of community		
Rural	194	89.8
Urban	22	10.2
Belongs to indigenous groups		
Yes	138	63.9
No	78	36.1
Education level		
Complete elementary school	8	3.7
Incomplete elementary school	6	2.8
Complete middle school	53	24.5
Incomplete middle school	5	2.3
Complete high school	91	42.1
Incomplete high school	18	8.3
Technical terminal with complete high school education	7	3.2
Technical terminal with incomplete high school education	2	0.9
Full degree	20	9.3
Incomplete bachelor's degree	4	1.9
Postgraduate studies	1	0.5
Technical terminal with complete mid school education	1	0.5
Occupation/occupations		
Housewife	196	90.7
Student	14	6.5
Unspecified occupations	3	1.4
Employed	3	1.4
Pregnancy number		
1 pregnancy	60	27.8
2 pregnancies	64	29.6
3 pregnancies	62	28.7
4 pregnancies	24	11.1
5 pregnancies	5	2.3
6 pregnancies	1	0.5
Age at first child		
10-19 years	108	50
20-29 years	90	41.7
30-39 years	14	6.5
40-49 years	4	1.9
Planned pregnancy		
Yes	32	14.8
No	184	85.2
Prenatal care		
Yes	216	100
No	0	0
Pregnancy complications		
Yes	73	33.8
No	143	66.2

With respect to marital status, the data indicated that 70.4% of the subjects were in a common-law relationship, 23.1% were married, 6% were single, and 0.5% were widowed.

A significant majority of the participants, 89.8% of the total sample (n=194), resided in rural communities, while 10.2% (n=22) inhabited urban areas. A similar proportion of the sample was comprised of indigenous peoples, with 63.9% (n=138) of the total sample belonging to this demographic.

Of the 216 patients surveyed, 24.5% (n=53) had completed secondary school, 42.1% (n=91) had completed high school, and 9.3% had completed a bachelor's degree. A mere 0.5% (n=1) of the participants had obtained postgraduate qualifications, with the remaining proportion distributed across the spectrum of other extant academic degrees, both completed and uncompleted (Table 1). Notwithstanding the aforementioned data, 90.7% (n=196) of patients were engaged in housework, 6.5% (n=14) were students, and the remainder (2.8%) were employed or engaged in unspecified activities.

With respect to the number of pregnancies, the majority of patients had between one and three pregnancies, with no significant differences in their distribution (27.8%, one pregnancy; 29.6%, two pregnancies; 28.7%, three pregnancies).

According to the patients' responses, 50% (n=108) had their first child between the ages of 10 and 19, 41.7% (n=90) between the ages of 20 and 29, 6.5% (n=14) between the ages of 30 and 39, and only 1.9% (n=4) between the ages of 40 and 49.

With regard to the planning phase, 184 (85.2%) patients reported no prior intention to conceive, yet it should be noted that all subjects were part of a prenatal care program.

Among the total number of patients surveyed, 66.2% (n=143) reported that they had not encountered any complications during their pregnancy, while the remaining 33.8% (n=73) indicated that they had experienced at least one complication.

The results for each item on the WCOCI were evaluated (Table 2). The initial finding indicated that, in general, all women received non-violent treatment from healthcare personnel. With respect to the matter of informed consent, it was reported by 81.9% (n=177) of patients that they were not provided with a consent form to sign upon admission to the hospital, and only 18.1% (n=39) reported having signed one.

**Table 2:** Results by item of the Woman-Centered Obstetric Care Index Source: Own elaboration

Item	Frequency	%
Nonviolent treatment		
Yes	216	100
No	0	0
Informed consent		
Yes	39	18.1
No	177	81.9
Authorization before each vaginal examination		
Yes	134	62
No	82	38
Procedures explanation		
Yes	147	68.1
No	69	31.9
Respectful treatment		
Yes	187	86.6
No	29	13.4
Accompaniment by a family member		
Yes	114	52.8
No	102	47.2
Enema and trichotomy avoided		
Yes	27	12.5
No	189	87.5
Close monitoring		
Yes	171	79.2
No	45	20.8
Care in intimacy		
Yes	128	59.3
No	88	40.7
Episiotomy and Kristeller avoided		
Yes	208	96.3
No	8	3.7
Postpartum indications		
Yes	216	100
No	0	0
Appropriate time		
Yes	140	64.8
No	76	35.2

Among the total number of female participants (n=216), 62% (n=134) were requested to provide consent prior to each vaginal examination, while 38% (n=82) were not requested to provide consent for the performance of this procedure.

Of the participants, 31.9% (n=69) indicated that they did not receive adequate explanations from healthcare personnel prior to each procedure (e.g., catheter placement, peripheral venous access, and medication administration). Conversely, 68.1% (n=147) of the participants reported receiving explanations before any procedure.

In addition to inquiries regarding violence-free care, respondents were also asked to evaluate the respect of rights and the absence of prejudice in care. Of the respondents, 86.6% (n=187) reported positive experiences with respect to this issue. On this same issue, 13.4% (n=29) of the respondents expressed a negative response.

A subsequent analysis was conducted to ascertain whether practices such as rectal enemas and trichotomy were avoided. The results indicated that 87.5% (n=189) of patients reported undergoing one or both of these practices, while only 12.5% (n=27) reported avoiding them.

Of the subjects, 79.2% (n=171) indicated that they had been the subject of close monitoring by healthcare personnel, while 20.8% (n=45) responded that this was not the case.

With respect to privacy concerns during their stay, 128 (59.3%) patients responded affirmatively; however, a substantial proportion of 88 (40.75%) patients responded that they did not feel safe or adequately cared for.

It is noteworthy that in 96.3% of cases, maneuvers such as Kristeller and routine episiotomy were avoided, and these procedures were only performed in 3.7% of cases.

Conversely, a uniform practice was observed in 100% of cases (n=216), wherein healthcare personnel consistently provided patients with postpartum or post-cesarean instructions. A significant proportion of patients, 64.8% (n=140), expressed satisfaction with the time allotted for their care, without perceiving any undue urgency or interventions intended to expedite the process.

An evaluation of the results of the Woman-Centered Obstetric Care Index (WCOCI) revealed that the majority of patients received care that was rated as "good," with this group accounting for 49.5% (n=107) of the total sample. Subsequently, the group of patients who received an "excellent" rating was identified, accounting for 38% (n=82) of the total percentage. Finally, 12.5% (n=27) of patients received "fair" care (Table 3).

**Table 3:** WCOCI classification WCOCI: Woman-Centered Obstetric Care Index

Classification	Frequency	%
Fair	27	12.5
Good	107	49.5
Excellent	82	38

The final classification yielded the following results: 87.5% (n=189) of patients received adequate care, while 12.5% (n=27) received inadequate care (Table 4).

**Table 4:** Final result of WCOCI WCOCI: Woman-Centered Obstetric Care Index

Final result	Frequency	%
Adequate care	189	87.5
Inadequate care	27	12.5

Prevalence ratios (PRs) were estimated using Poisson models with robust variance, with 95% confidence intervals (CIs). The factors associated with adequate care were found to be as follows: close monitoring (PR=1.72, 95% CI: 1.30-2.28), explanation of procedures (PR=1.55, 95% CI: 1.20-2.00), prior authorization for vaginal examination (PR=1.40, 95% CI: 1.05-1.87), accompaniment by a family member (PR=1.32, 95% CI: 1.01-1.70), and respect for the time needed (PR=1.28, 95% CI: 1.00-1.65). The values are presented in Table 5.

**Table 5:** PR: factors associated with adequate care PR: prevalence ratio, CI: confidence interval

Factor	PR	95% CI
Close monitoring	1.72	1.30-2.28
Procedures explanation	1.55	1.20-2.00
Authorization before each vaginal examination	1.40	1.05-1.87
Accompaniment by a family member	1.32	1.01-1.70
Appropriate time	1.28	1.00-1.65

Following the implementation of the Kolmogorov-Smirnov normality test, the level of correlation between each of the points of good medical practice and the final WCOCI result was calculated using Pearson's formula. The most salient correlation coefficients were identified for the items "close monitoring" (r=0.737), "explanation of procedures" (r=0.552), and "authorization before each physical examination" (r=0.425). In a similar vein, although with weaker values, the items "accompaniment by a family member" (r=0.343) and "appropriate time" (r=0.337) were also identified (Table 6).

**Table 6:** Pearson correlation between items and the final WCOCI score WCOCI: Woman-Centered Obstetric Care Index

Item	WCOCI score
Close monitoring	0.737
Procedures explanation	0.552
Authorization before each vaginal examination	0.425
Accompaniment by a family member	0.343
Appropriate time	0.337

## Discussion

Our findings suggest that, although most women were classified as receiving adequate care, key gaps persisted in domains central to respectful maternity care, particularly written informed consent at admission and perceived privacy during care. These results are consistent with prior evidence indicating that consent-related failures are among the most common forms of mistreatment. However, direct comparisons with population-based surveys should be interpreted cautiously because definitions, measurement approaches, and recall periods differ [8,9].

A study by Talya et al. revealed a correlation between feelings of being overworked and an elevated prevalence of disorders, encompassing physical, emotional, and psychological facets [10]. The investigation, which involved a sample of 109 medical residents, found that sleep deprivation and work overload after 24 hours exerted a deleterious effect on concentration, visual attention, and gait.

In the study sample, among healthcare personnel, it was found that a total of four midwives collaborated with healthcare professionals in the care of pregnant women. This finding is of particular significance as it aligns with the recommendations set forth by the Committee on the Elimination of Discrimination against Women in 2018 [11,12]. The committee asserts that collaboration with traditional midwives and the training of healthcare personnel in rural areas contribute to a reduction in the incidence of maternal mortality. It is noteworthy that the findings of our research reflect only limited adherence to this aspect, as evidenced by the fact that among the entire sample of staff interviewed, a mere 5.8% have undergone training related to obstetric violence within the past two years. While the absence of this information is regrettable, it is imperative that this knowledge be utilized as an area of opportunity in future evaluations of healthcare personnel and upcoming implementations of strategies that reinforce the quality of care.

The aforementioned factors have been identified as fundamental components of optimal medical practice, which play a pivotal role in the delivery of care. Consequently, it is imperative that healthcare professionals accord these factors the necessary attention and consideration. A similar conclusion was reported in a 2019 study by da Silva Carvalho and Brito, which evaluated 314 postpartum women and analyzed various practices [12]. The study found that allowing a family member to accompany the patient and answering questions clearly were the practices most closely related to providing good care [13,14].

Although, in general, it was found that most women receive adequate obstetric care, it is clear that certain practices still exist that reduce the quality of care and compromise the individual integrity of patients. Although current efforts to reduce this situation have been successful, existing cases may go unnoticed. As Rodríguez Mir et al. explain, the reasons for this often range from inadequate professional training to patients' lack of knowledge on the subject [15].

Limitations

Although categorizing obstetric care from the perspective of patients and healthcare personnel reduces the subjective bias that each patient's opinion may have, the cross-sectional nature of the study could limit the reliability of the final categorization of care, as it even covers moments prior to hospital admission, such as care in the emergency room.

One point that could generate bias is the convenient selection of healthcare professionals included in the study. Similarly, the lack of actual measurement of patients' income could generate bias, as the data recorded were provided verbally and were not supported by a document verifying their income. This means that relying on data reported directly by healthcare professionals and patients, rather than being evaluated at the time of care or verified beyond the time of the study, could lead to omissions in the questions asked and bias in the results.

Similarly, the final proposed categorization (adequate care/inadequate care) could lead to the omission of specific points of care from the WCOCI score.

## Conclusions

In the Nacajuca health network, postpartum women were predominantly classified as receiving adequate woman-centered obstetric care according to the Woman-Centered Obstetric Care Index (WCOCI). The results also highlight specific, modifiable process domains, particularly strengthening standardized informed consent procedures and reinforcing privacy safeguards, that represent high-yield opportunities to further consolidate respectful maternity care within routine clinical practice.

## Appendices

Instrument: Woman-Centered Obstetric Care Index (WCOCI)

The primary measurement instrument used in this study was the Woman-Centered Obstetric Care Index (WCOCI), developed ad hoc to assess, from the perspective of postpartum women, the extent to which intrapartum and immediate postpartum care aligned with the principles of woman-centered obstetric care and respectful maternity care, as endorsed by international quality of care frameworks and national clinical guidelines.

The WCOCI operationalizes a set of key practices of respectful care and the absence of routine interventions without a clear clinical indication. It comprises 12 dichotomous items that capture core dimensions of dignified treatment, communication, informed consent, companionship, privacy, close clinical monitoring, and respect for the physiology of labor and birth.

Each item is answered with the options “Yes” or “No,” coded as follows: 1 point = the practice was present/fulfilled and 0 points = the practice was absent/not fulfilled.

Higher scores indicate a higher degree of woman-centered obstetric care.

The 12 items that compose the WCOCI are as follows:

1. Non-violent care: "During my care, I received treatment free of violence from health personnel."

2. Informed consent at admission: "At hospital admission, I was asked to sign an informed consent document."

3. Authorization before each vaginal examination: "Before each vaginal examination, staff asked for my authorization."

4. Explanation of procedures: "Health personnel explained in advance the procedures that were going to be performed (e.g., placement of catheters, intravenous lines, administration of medications, or others)."

5. Respectful and non-discriminatory treatment: "I felt treated with respect and without prejudice throughout my care."

6. Companionship by a family member: "I was allowed to be accompanied by a family member of my choice during part of labor or the immediate postpartum period."

7. Avoidance of routine enema and perineal shaving: "I did not receive an enema or perineal shaving as a routine measure, without clear explanation or indication."

8. Close clinical monitoring: "I felt that health personnel closely monitored my progress and that of my baby."

9. Privacy and intimate care: "I felt that my privacy was respected (use of curtains or screens, avoiding unnecessary exposure of my body)."

10. Avoidance of routine Kristeller maneuver and episiotomy: "The Kristeller maneuver was not applied, and an episiotomy was not performed as a routine measure, without a justified medical reason."

11. Postpartum/post-cesarean instructions: "At discharge, I received clear instructions regarding postpartum/post-cesarean care and warning signs."

12. Adequate time for care: "I consider that the necessary time for my care was respected, without undue pressure to accelerate the process."

The total WCOCI score is obtained by summing the 12 items, yielding a possible range from 0 to 12 points. Based on the distribution of scores and expert consensus regarding the clinical relevance of the practices, four ordinal quality categories were defined: 0-3 points = "poor" obstetric care (clearly inadequate), 4-7 points = "fair" obstetric care (partially inadequate), 8-10 points = "good" obstetric care (adequate), and 11-12 points = "excellent" obstetric care (highly adequate and woman-centered).

For the main analyses, these categories were collapsed into a dichotomous outcome variable: adequate obstetric care = "good" and "excellent" categories, and inadequate obstetric care = "poor" and "fair" categories.

This dichotomization allows the estimation of prevalence ratios of adequate care according to the presence or absence of specific woman-centered practices and the sociodemographic and obstetric characteristics of women and health personnel.

Psychometric validation of the WCOCI

Psychometric validation of the WCOCI was conducted in the same sample of postpartum women included in the main study. The process comprised the assessment of content and face validity, internal consistency, and sampling adequacy for factorial analyses.

Content validity was ensured through review of the instrument by an expert panel including obstetricians, obstetric nurses, public health specialists, and human rights experts in maternal health. The panel evaluated the pertinence, relevance, and clarity of each item in relation to the construct "woman-centered obstetric care," as well as its consistency with standards of respectful maternity care and national guidelines. Expert feedback was used to refine item wording and to ensure coverage of key dimensions of dignified treatment, communication, informed consent, companionship, privacy, and non-abusive clinical practices.

Face validity was explored through a pilot test with a small group of postpartum women from the same setting, who completed the preliminary WCOCI and provided feedback on comprehension, clarity of language, and time required for completion. Minor wording adjustments were made to improve comprehensibility without altering the substantive content of the items.

The internal consistency of the WCOCI was evaluated using Cronbach's alpha coefficient. In the final sample of 216 women, the index showed a Cronbach's alpha of 0.82, indicating good internal consistency for a 12-item dichotomous scale. No item substantially increased the overall alpha when deleted; therefore, all 12 items were retained. These findings suggest that WCOCI items coherently measure a common underlying construct related to woman-centered obstetric care.

The correlation between each item and the total WCOCI score was also examined. The items with the strongest correlations with the global score were "close clinical monitoring," "explanation of procedures," and "authorization before each vaginal examination," followed by "companionship by a family member" and "adequate time for care." These patterns support the internal validity of the index, highlighting that practices related to monitoring, communication, and informed consent contribute substantially to variability in the overall score.

Sampling adequacy for assessing the internal structure of the WCOCI was evaluated using the Kaiser-Meyer-Olkin (KMO) index and Bartlett's test of sphericity. The KMO value was 0.78, which is considered acceptable for exploratory factor analyses, and Bartlett's test was statistically significant (p<0.001), indicating that the correlation matrix departs from identity and is suitable for identifying latent factors. Although a full exploratory or confirmatory factor analysis was beyond the scope of this study, these results support the coherence of the index and its potential for more detailed psychometric analyses in future research.

Statistical analysis

All statistical analyses were conducted using IBM SPSS Statistics version 27 (IBM Corp., Armonk, NY). The analytical strategy comprised three levels: descriptive, bivariate, and multivariable analyses, considering adequate obstetric care according to the WCOCI as the primary outcome.

Descriptive analyses included the distribution of sociodemographic and obstetric characteristics of postpartum women, as well as key labor and birth characteristics and core features of health personnel. Categorical variables were summarized as absolute and relative frequencies, whereas continuous variables were summarized using measures of central tendency and dispersion (mean and standard deviation, or median and interquartile range, as appropriate). For the WCOCI, the distribution of the total score (0-12), the proportion of women in each ordinal quality category ("poor," "fair," "good," and "excellent"), and the overall proportion of adequate versus inadequate care were described.

Bivariate analyses were used to explore associations between woman-centered obstetric care and the independent variables of interest. The dichotomous WCOCI outcome (adequate versus inadequate care) was cross-tabulated with each WCOCI item and with selected sociodemographic and obstetric variables. Pearson's chi-squared tests were applied to assess statistical significance, and crude prevalence ratios (PRs) with 95% confidence intervals (95% CIs) were estimated, using the absence of the practice (WCOCI item = 0) as the reference category.

To identify factors independently associated with adequate obstetric care, multivariable analyses were performed using Poisson regression models with robust variance. These models are appropriate for estimating prevalence ratios in cross-sectional studies with common outcomes. The dependent variable was the dichotomous indicator of adequate obstetric care derived from the WCOCI (1 = adequate, 0 = inadequate). Independent variables included key woman-centered practices (WCOCI items) and sociodemographic and obstetric covariates considered potential confounders (such as maternal age, marital status, belonging to an indigenous population, and mode of delivery).

Adjusted prevalence ratios (aPRs) with 95% CIs and p-values were reported. Variable selection in the multivariable models was guided by theoretical considerations based on the multilevel framework of determinants of obstetric care and by the patterns observed in bivariate analyses, avoiding overfitting given the sample size and the frequency of the outcome. Statistical significance was set at α=0.05 for all analyses.
